# Population structure and molecular genetic characterization of 5-flucytosine-susceptible and -resistant clinical *Candida dubliniensis* isolates from Kuwait

**DOI:** 10.1371/journal.pone.0175269

**Published:** 2017-04-05

**Authors:** Mohammad Asadzadeh, Suhail Ahmad, Noura Al-Sweih, Ziauddin Khan

**Affiliations:** Department of Microbiology, Faculty of Medicine, Kuwait University, Safat, Kuwait; Louisiana State University, UNITED STATES

## Abstract

*Candida dubliniensis* and *Candida albicans* are two closely related species. Although *C*. *dubliniensis* is less pathogenic, it has a higher propensity to develop resistance to fluconazole and some strains exhibit intrinsic resistance to 5-flucytosine (5-FC). All 5-FC-resistant isolates from Kuwait were previously shown to belong to one of seven internal transcribed spacer (ITS) region of rDNA-based haplotypes. This study performed fingerprinting of *C*. *dubliniensis* isolates by multilocus sequence typing (MLST) to determine population structure of 5-FC-resistant and -susceptible strains and compared the results with data from a global collection of isolates. Fifty-two *C*. *dubliniensis* isolates previously analyzed and 58 additional isolates mostly collected during 2010–2013 and characterized by phenotypic and molecular methods were used. ITS-based haplotypes were identified by haplotype-specific PCR and/or by PCR-DNA sequencing of rDNA. Population structure was determined by 8-loci-based MLST. E-test was used to determine susceptibility to 5-FC, fluconazole, voriconazole and amphotericin B. Five ITS haplotypes (ITSH) were detected among 110 *C*. *dubliniensis* isolates. The ITSH1 was most common (n = 80 isolates) followed by ITSH4 (n = 25 isolates). Two isolates each belonged to ITSH5 and ITSH8 while one isolate belonged to ITSH7. MLST identified 16 diploid sequence types (DSTs) including six new DSTs. DST11 (n = 52) and DST14 (n = 25) were dominant genotypes and were confined (together with DST21) to Middle-Eastern countries. Other DSTs (excluding some new DSTs) had a wider global distribution as they were identified from various other countries. Only ITSH4 isolates (n = 25) belonged to DST14, were resistant to 5-FC and contained S29L mutation in Cd*FCA*1. ITSH5, ITSH7 and ITSH8 isolates belonged to different DSTs. Thus, clinical *C*. *dubliniensis* isolates in Kuwait exhibited limited genotypic heterogeneity and most isolates belonged to region-specific DSTs. All 5-FC-resistant *C*. *dubliniensis* isolates belonged to ITSH4 and MLST-based DST14 genotype. Placement of some isolates into additional ITS haplotypes is also supported by MLST data.

## Introduction

*Candida dubliniensis* and *Candida albicans* are the only two yeast species capable of forming true hyphae which play an important role in adhesion and tissue invasion during infection [1]. Although both species share many phenotypic and genotypic characteristics, *C*. *dubliniensis* is less pathogenic, likely as a result of significant gene loss since their divergence from the common ancestor [1]. However, *C*. *dubliniensis* also exhibits a worldwide distribution, is increasingly being isolated from clinical specimens and its role as a bloodstream pathogen appears to be underestimated as it was recorded as the fourth most common *Candida* spp. causing bloodstream infections in some studies [2–12]. Furthermore, *C*. *dubliniensis* exhibits increased adherence to buccal epithelial cells, a higher propensity to form biofilms than *C*. *albicans* and reduced susceptibility to azole antifungal drugs [2, 9, 13–16].

Previous studies have investigated the population structure of *C*. *dubliniensis* using fingerprinting with species-specific repeat sequence-containing molecular probes [17, 18]. Fingerprinting with Cd25 probe detected three distinct clades, Cd25 group I, Cd25 group II and Cd25 group III among *C*. *dubliniensis* isolates [17, 18]. Further studies based on sequencing of the internal transcribed spacer (ITS) region (including ITS1-5.8S rRNA-ITS2) of rDNA identified 4 distinct genotypes/haplotypes where ITS haplotype (ITSH)1 and ITSH2 isolates exhibited global distribution while ITSH3 and ITSH4 isolates were mainly confined to two middle Eastern countries (Egypt and Saudi Arabia) and most (17 of 20) isolates were resistant to 5-flucytosine (5-FC) [18, 19]. Although *C*. *dubliniensis* exhibits greater karyotype variability than *C*. *albicans*, the population structure of a global collection of *C*. *dubliniensis* isolates by multilocus sequence typing (MLST) based on 8–10 protein-coding gene fragments was found to be less divergent than *C*. *albicans* as it comprised only three distinct clades (C1–C3) and all 5-FC-resistant isolates were confined to MLST clade C3, which also included some 5-FC susceptible isolates [20]. Although 7–10 protein-coding gene fragments were initially used in different combinations during development of the MLST scheme, a combination of 8 loci (Cd*AAT1b*, Cd*ACC1*, Cd*ADP1*, Cd*MPIb*, Cd*RPN2*, Cd*SYA1*, exCd*VPS13* and exCd*ZWF1b*) was recommended for the MLST of *C*. *dubliniensis* isolates on the basis of the highest number of genotypes per variable base [20].

In our previous study based on 103 clinical *C*. *dubliniensis* isolates from Kuwait [21], we found 68 ITSH1 and 25 ITSH4 isolates and five additional haplotypes (labeled as ITSH5 to ITSH9) but failed to detect any ITSH2 or ITSH3 isolates, described previously by Gee et al. [18]. The significance of these haplotypes among clinical *C*. *dubliniensis* isolates is not entirely clear at present. Interestingly, the ITS region sequence of our ITSH5 isolates matched with a 5-FC-resistant strain (Eg207) which was isolated from Egypt but it was labeled as a variant of haplotype 4 (haplotype 4A) while the sequence of our ITSH7 isolate matched with a 5-FC-susceptible strain (Is35) which was isolated from Israel and it was also labeled as a variant of haplotype 4 (haplotype 4B) rather than as a new haplotype [18, 21]. On the other hand, all 25 of our 5-FC-resistant *C*. *dubliniensis* isolates from Kuwait belonged to ITSH4 only while all other isolates were susceptible to 5-FC including ITSH5 isolates even though they differed at only one nucleotide position within the ITS region from ITSH4 isolates [21]. However, several questions remained unanswered at that time. For instance, it was not known whether additional ITS haplotypes detected in our previous study are also supported by MLST and whether all 5-FC resistant isolates belonging to ITSH4 belong to one or more MLST sequence types. Only one previous study has been carried out to explore the population structure of clinical *C*. *dubliniensis* strains [20]. However, the clinical isolates included in that study comprised a global collection of epidemiologically unrelated strains isolated at diverse geographical locations [20]. Thus, there was a need to carryout fingerprinting of clinical *C*. *dubliniensis* isolates from a single country and to compare the results with data obtained previously with the global collection of isolates.

This study was carried out to determine the population structure of *C*. *dubliniensis* strains isolated at a single geographical location by MLST and to correlate the MLST sequence types with ITS haplotype and resistance to 5-FC and other (fluconazole, voriconazole and amphotericin B) antifungal drugs. The phylogenetic relationship of clinical *C*. *dubliniensis* isolates from Kuwait was further studied by comparing our MLST data with a global collection of isolates analyzed previously by MLST to better understand the population structure of *C*. *dubliniensis*.

## Materials and methods

### Reference strains and clinical isolates of *C*. *dubliniensis*

Reference strains of *C*. *dubliniensis* (CD36), *C*. *albicans* (ATCC 90028) and *C*. *parapsilosis* (ATCC 22019) were used. Although all 103 *C*. *dubliniensis* isolates analyzed in our previous study were stored as frozen cultures, only 52 isolates were available for this study as the remaining isolates, unfortunately, could not be revived from frozen cultures. Consequently 58 additional *C*. *dubliniensis* isolates recovered from various clinical specimens were also included. While 16 of these additional isolates were recovered during the same time period (2002 to 2010) as the previous 52 isolates, the remaining 42 isolates were recovered during 2011–2013. Most (90 of 110) of these isolates originated from oral cavity/respiratory system specimens including sputum (n = 59), tracheal/endotracheal aspirate (n = 19), oral/throat swab (n = 8) and bronchoalveolar lavage (n = 4). The remaining 20 isolates were recovered from other specimen types which included urine (n = 6), vaginal swab (n = 6), drained fluid (n = 4), catheter tip (n = 2) and one isolate each from blood and wound swab. Thus, a total of 110 *C*. *dubliniensis* isolates recovered from various clinical specimens of 110 individual patients were used in the present study. The clinical specimens were collected at various hospitals across Kuwait after obtaining verbal consent from patients as part of routine patient care for the isolation of fungal pathogens. The isolates were cultured on Sabouraud dextrose agar medium in clinical microbiology laboratories of various hospitals in Kuwait. The isolates were then sent to Mycology Reference Laboratory, Department of Microbiology, Kuwait University for identification and antifungal susceptibility testing and data were analyzed anonymously. The consent procedure and the study were approved by the Joint Committee for the Protection of Human Subjects in Research, Health Sciences Center, Kuwait University and Ministry of Health, Kuwait. The detailed list of all 110 *C*. *dubliniensis* isolates with their ITS haplotype, diploid sequence type (DST), amino acid at *CdFCA1* codon 29 and accession numbers of representative DNA sequences submitted to EMBL/GenBank databases for different ITS haplotypes/new diploid sequence types (DST27-DST33) detected in this study and for *CdFCA1* codon 29 region is provided in S1 Table.

All *C*. *dubliniensis* isolates were identified by their assimilation profiles on Vitek2 yeast identification system (bioMérieux, Marcy-l’Etoile, France) and by their ability to form germ tubes in serum and by their specific phenotypic characteristics on sunflower seed agar medium, as described previously [22, 23]. The identity was further confirmed by extraction of genomic DNA from each isolate and species-specific amplification of ITS region of rDNA. The genomic DNA was extracted from 1 ml of cell suspension by using Gentra Puregene Yeast DNA extraction kit (Qiagen, Hilden, Germany) according to kit instructions. The duplex PCR was performed by using (CDUF, 5’-AAACTTGTCACGAGATTATTTTT-3’; CDUR, 5’-AAAGTTTGAAGAATAAAATGGC-3’; CALF, 5′-TGGTAAGGCGGGATCGCTT-3′ and CALR, 5′-GGTCAAAGTTTGAAGATATAC) primers and the reaction and PCR cycling conditions as described previously [24]. The amplicons were detected by agarose gel electrophoresis, performed as described previously [25].

### Identification of ITS haplotypes by PCR amplification

The ITS haplotype of each *C*. *dubliniensis* isolate was determined by using haplotype-specific primers for ITSH1 to ITSH4 and the reaction and PCR cycling conditions as described previously [21]. The amplified products (10 μl) were resolved by electrophoresis in 2% (wt/vol) agarose gels and presence of a single amplicon of the expected size indicated the specific haplotype. For isolates yielding an amplicon with more than one haplotype-specific primer pair, the ITS haplotype was determined by PCR-DNA sequencing of the ITS region of rDNA. The complete ITS region (containing ITS-1, 5.8S rRNA and ITS-2) of rDNA was amplified by using panfungal ITS1 and ITS4 primers, as described previously [26]. The amplicons were purified by using a PCR product purification kit (Qiagen, Hilden, Germany) used according to kit instructions. Both strands of purified amplicons were sequenced. Sequencing reactions were carried out by using ITS1FS, ITS2, ITS3 and ITS4RS as sequencing primers by using BigDye terminator v3.1 cycle sequencing kit and ABI 3130*xl* Genetic Analyzer by following manufacturer’s instructions (Applied Biosystems Inc.) and as described previously [27]. Pair-wise comparisons with sequences of specific haplotypes were carried out by using Clustal omega. The sequence data for various ITS haplotypes have been submitted to European Molecular Biology Laboratory (EMBL) database under accession numbers LT608348 to LT608369 and LT716018 to LT716020.

### Population structure of *C*. *dubliniensis* isolates by MLST

All 110 *C*. *dubliniensis* isolates were genotyped by using the MLST scheme based on PCR amplification and DNA sequencing of eight recommended housekeeping gene (Cd*AAT1b*, Cd*ACC1*, Cd*ADP1*, Cd*MP1b*, Cd*RPN2*, Cd*SYA1*, *exCdVPS13* and *exCdXWF1b*) fragments that yielded the highest number of genotypes per variable base as described by McManus et al. [20]. The PCR amplification and cycling condition were same as described previously [20]. The amplicons were purified and subjected to bi-directional sequencing as described above except that gene-specific primers used for amplification of each housekeeping gene fragment were also used as sequencing primers [20]. Sequence data generated by the ABI 3130xl genetic analyzer were checked for confidence levels with an ABI sequence scanner, reverse compliments were generated and aligned with forward sequences using Clustal omega. The sequences of various loci were assembled, the locus-specific diploid sequence type (locus-DST) for eight housekeeping genes were assigned and the combined locus-DSTs defined the final diploid sequence type (DST) for a given isolate, as described previously [20]. Since a publicly available online curated MLST database similar to *C*. *albicans* is not available for *C*. *dubliniensis*, additional DSTs detected in this study were assigned a higher number (DST27-DST33) considering that only 26 DSTs (based on the analysis of 10 housekeeping gene fragments instead of eight housekeeping gene fragments recommended by McManus et al. [20] and used in this study) were detected previously. The concatenated sequence data for 8 loci (Cd*AAT1b*, Cd*ACC1*, Cd*ADP1*, Cd*MP1b*, Cd*RPN2*, Cd*SYA1*, exCd*VPS13* and exCd*XWF1b*) have been submitted to EMBL database under accession numbers LT608387 to LT608393.

Based on MLST profile obtained from *C*. *dubliniensis* isolates, a dendrogram was constructed by using BioNumerics version 7.5 (Applied Maths, Sint-Martens-Latem, Belgium) using standard unweighted pair group method with arithmetic mean (UPGMA) settings. The *C*. *dubliniensis* isolates from Kuwait were considered as belonging to the same DST when they contained the same locus-specific DSTs for all eight loci. Only one isolate was used as a representative for each DST for determining phylogenetic relationships. The DST data were also compared with those obtained from a global collection of 50 epidemiologically unrelated *C*. *dubliniensis* isolates analyzed previously [20]. For this purpose, the MLST profiles for each of 50 *C*. *dubliniensis* isolates were obtained from the published data [20].

### Antifungal drug susceptibility testing

The susceptibility of *C*. *dubliniensis* isolates against antifungal drugs, amphotericin B (AP), 5-flucytosine (5-FC), fluconazole (FL) and voriconazole (VO) was determined by E-test (AB Biodisk, Solna, Sweden) on RPMI agar (supplemented with 2% glucose and buffered with MOPS, 0.165 M, pH 7.0) plates according to manufacturer’s recommendations and as described previously [28]. Briefly, the E-test was performed by preparing a homogenized suspension of each isolate in 2 ml of sterile normal saline and the turbidity was adjusted to 0.5 McFarland standard. The plates were inoculated uniformly with cotton swabs, E-test strips were applied, the plates were incubated at 35 °C and read after 24 h. The minimum inhibitory concentration (MIC) values (mg/L) were recorded for each drug and MIC50 and MIC90 values were also calculated. The revised interpretive susceptibility breakpoints as recommended by Clinical Laboratory Standards Institute (CLSI) were used for FL (≤2 mg/L, susceptible; 4 mg/L, susceptible dose-dependent and ≥8 mg/L, resistant), 5-FC (≤4 mg/L, susceptible; 8–16 mg/L, susceptible dose-dependent and ≥32 mg/L, resistant) and VO (≤0.125 mg/L, susceptible; 0.25–0.5 mg/L, susceptible dose-dependent and ≥1 mg/L, resistant) [29]. Due to lack of defined breakpoints for AP, an isolate showing MIC ≤1.0 mg/L was considered as susceptible. Quality control was ensured by testing *C*. *albicans* ATCC 90028 and *C*. *parapsilosis* ATCC 22019, as recommended by CLSI.

### Detection of 5-flucytosine resistance-conferring mutations in cytosine deaminase

It has previously been shown that resistance of *C*. *dubliniensis* isolates to 5-FC is due to a missense mutation (S29L) at codon 29 of cytosine deaminase encoded by Cd*FCA1* gene [30]. The Cd*FCA1* gene region around codon 29 was amplified by PCR by using FCA1F (5′-ATCACGATGACATTTGACGACAA-3 and FCA1R (5′-CATACTACATGGTGACAAAGTAGT-3′) primers and PCR reaction and cycling conditions as described previously [21]. The amplicons were purified and subjected to restriction digestion with Dpn II restriction enzyme to generate restriction fragment length polymorphism (RFLP) indicating the presence of serine or leucine at codon 29 or sequenced with FCA1FS (5′-TTTGACGACAAAAAAGGTTTACA-3′) and FCA1RS (5′-ATTTCCCCATGTAAAATAGATGA-3′) as sequencing primers for the detection of mutations at codon 29 of Cd*FCA1* gene, as described previously [21]. The Cd*FCA1* sequencing was performed to confirm the results of RFLP for 75 *C*. *dubliniensis* isolates including 45 of 80 ITSH1 isolates and all isolates (n = 30) belonging to ITSH4, ITSH5, ITSH7 and ITSH8. Specific nucleotide/amino acid substitutions were identified by DNA and amino acid sequence alignments of Cd*FCA1* gene/encoded protein from 5-FC-susceptible and 5-FC-resistant *C*. *dubliniensis* isolates by using Clustal omega [21]. The DNA sequence data for wild-type and mutant Cd*FCA1* sequences have been submitted to EMBL/GenBank databases under accession numbers LT608370 to LT608386 and KX809941 to KX809946. The selected *C*. *dubliniensis* isolates have been submitted to Centraalbureau voor Schimmelcultures (CBS) under accession numbers CBS 14716 to CBS 14719.

## Results

### ITS haplotypes among *C*. *dubliniensis* isolates

Among the 52 *C*. *dubliniensis* isolates analyzed previously, 39, 10, one, one, and one isolate belonged to ITSH1, ITSH4, ITSH5, ITSH7 and ITSH8, respectively [21]. Based on haplotype-specific PCR amplification and/or DNA sequencing studies, 41, 15, one and one isolate belonged to ITSH1, ITSH4, ITSH5 and ITSH8, respectively, among 58 *C*. *dubliniensis* isolates additionally analyzed in this study. Similar to our previous study [21], ITSH2 or ITSH3 isolates were not detected again among *C*. *dubliniensis* isolates in Kuwait. Collectively, of the 110 *C*. *dubliniensis* isolates analyzed in this study, 80 (73%) isolates belonged to ITSH1, 25 (23%) belonged to ITSH4, two isolates each belonged to ITSH5 and ITSH8 and only one isolate belonged to ITSH7 (Table 1).

**Table 1:** Summary of fingerprinting data for 110 *C*. *dubliniensis* isolates using ITS-region sequencing, MLST and susceptibility to 5-flucytosine.

ITS-based haplotype	DST ^a^	No. of isolates	Locus-specific diploid sequence type profile of								Susceptibility to 5-flucytosine	Cd*FCA1* codon 29
			Cd*AAT1b*	Cd*ACC1*	Cd*ADP1*	Cd*MPIb*	Cd*RPN2*	Cd*SYA1*	exCd*VPS13*	exCd*ZWF1b*		
ITSH1	DST2	5	1	1	1	1	1	4	1	1	Susceptible	Wild-type
ITSH1	DST4	2	1	1	1	2	1	4	1	1	Susceptible	Wild-type
ITSH1	DST5	2	1	1	1	3	1	4	1	1	Susceptible	Wild-type
ITSH1	DST6	4	1	1	1	3	3	4	1	1	Susceptible	Wild-type
ITSH1	DST7	8	1	1	1	4	1	4	1	1	Susceptible	Wild-type
ITSH1	DST9	1	1	1	1	5	1	4	1	1	Susceptible	Wild-type
ITSH1	DST11	52	1	1	1	6	3	4	1	1	Susceptible	Wild-type
ITSH1	DST27	1	1	1	1	6	1	4	1	1	Susceptible	Wild-type
ITSH1	DST28	2	1	1	1	8^b^	3	4	1	1	Susceptible	Wild-type
ITSH1	DST29	1	1	1	6	4	1	4	1	1	Susceptible	Wild-type
ITSH1	DST30	1	1	1	6	6	3	4	1	1	Susceptible	Wild-type
ITSH1	DST31	1	6^b^	1	1	4	1	4	1	1	Susceptible	Wild-type
**ITSH4**	**DST14**	**25**	**1**	**2**	**6**	**2**	**1**	**2**	**3**	**4**	**Resistant**	**S29L**
ITSH5	DST21	2	3	3	1	2	1	2	3	5	Susceptible	Wild-type
ITSH7	DST4	1	1	1	1	2	1	4	1	1	Susceptible	Wild-type
ITSH8	DST33	1	1	3	1	4	1	4	1	1	Susceptible	Wild-type
ITSH8	DST32	1	1	3	1	1	1	4	1	1	Susceptible	Wild-type

^a^DST, diploid sequence type

^b^These are new alleles and the DNA sequences of these alleles are provided in S2 Table

### Antifungal susceptibility testing of *C*. *dubliniensis* isolates

The results of susceptibility testing for *C*. *dubliniensis* isolates to four antifungal agents are shown in Table 2. Nearly all *C*. *dubliniensis* isolates were susceptible to three; amphotericin B (AP), fluconazole (FL) and voriconazole (VO) antifungal agents except one isolate which showed reduced susceptibility to FL (MIC = 4 mg/L). The isolates exhibited MIC50 and MIC90 values of 0.008 and 0.047 mg/L for AP, 0.19 and 0.5 mg/L for FL, and 0.012 and 0.047 mg/L for VO, respectively. However, only 85 *C*. *dubliniensis* isolates were susceptible to 5-FC while 25 isolates (including 15 of 58 isolates analyzed for the first time during this study) were resistant to 5-FC (MIC ≥32 mg/L). Again, similar to our previous study, all 25 5-FC-resistant isolates belonged to ITSH4 only while all isolates belonging to ITSH1, ITSH5, ITSH7 and ITSH8 (including isolates of these haplotypes analyzed in this study) were susceptible to 5-FC.

**Table 2:** Minimum Inhibitory Concentration (MIC) ranges and susceptibility data for 110 *Candida dubliniensis* isolates by E-test.

Antifungal agent	MIC 50 (mg/L)	MIC 90 (mg/L)	Range (mg/L)	No. of isolates detected as		
				Susceptible	Susceptible dose-dependent	Resistant
Amphotericin B	0.008	0.047	0.002–0.75	110	0	0
5-Flucytosine	0.023	≥32	0.002–≥32	85	0	25
Fluconazole	0.19	0.5	0.008–4	109	1	0
Voriconazole	0.012	0.047	0.002–0.23	110	0	0

### Molecular basis of resistance to 5-FC in *C*. *dubliniensis* isolates

All *C*. *dubliniensis* isolates belonging to different ITS haplotypes (ITSH1, ITSH5, ITSH7 and ITSH8) that were susceptible to 5-FC contained wild-type sequence (TCA) at Cd*FCA1* codon 29. However, the 25 *C*. *dubliniensis* isolates belonging to ITSH4 and resistant to 5-FC (including 15 isolates analyzed for the first time in this study) contained a missense (TCA to TTA) mutation at Cd*FCA1* codon 29 leading to substitution of serine by leucine (S29L) in cytosine deaminase.

### Genetic diversity of *C*. *dubliniensis* isolates in Kuwait by MLST

All eight housekeeping gene fragments were successfully amplified by using the corresponding gene-specific forward and reverse primers and the sizes of PCR amplicons in each case were as described previously [20]. The assembled DNA sequences for each gene fragment were used for locus-specific diploid sequence type (locus-specific DST). The most informative gene was Cd*MPIb*, identifying seven locus-specific DSTs while the remaining loci were less polymorphic and less informative (Table 1). The combination of eight locus-specific DSTs yielded the final DST for each isolate. Based on these analyses, 110 *C*. *dubliniensis* isolates from Kuwait were classified into only 16 DSTs. Of 16 DSTs, nine DSTs have been described previously while seven DSTs (DST27 to DST33) were identified for the first time in this study (Table 1). Of the seven new DSTs, five DSTs (DST27, DST29, DST30, DST32 and DST33) were generated by rearrangement of previously known locus-specific DSTs while the remaining two DSTs (DST28 and DST31) were formed due to the presence of a new locus-specific DST for a single gene (Cd*MP1b* for DST28 and Cd*AAT1b* for DST31) in each case. The DNA sequences of the new alleles are provided in S2 Table. The two most common DSTs were DST11 and DST14, shared among 52 (47%) and 25 (23%) isolates, respectively (Table 1). More importantly, all 25 *C*. *dubliniensis* isolates resistant to 5-FC belonged to a single DST (DST14). The DNA sequences of the coding regions of eight housekeeping genes were also concatenated to generate the combined data set for each isolate and were submitted to GenBank/EMBL database.

### Population structure of *C*. *dubliniensis* isolates

**Fig 1:**
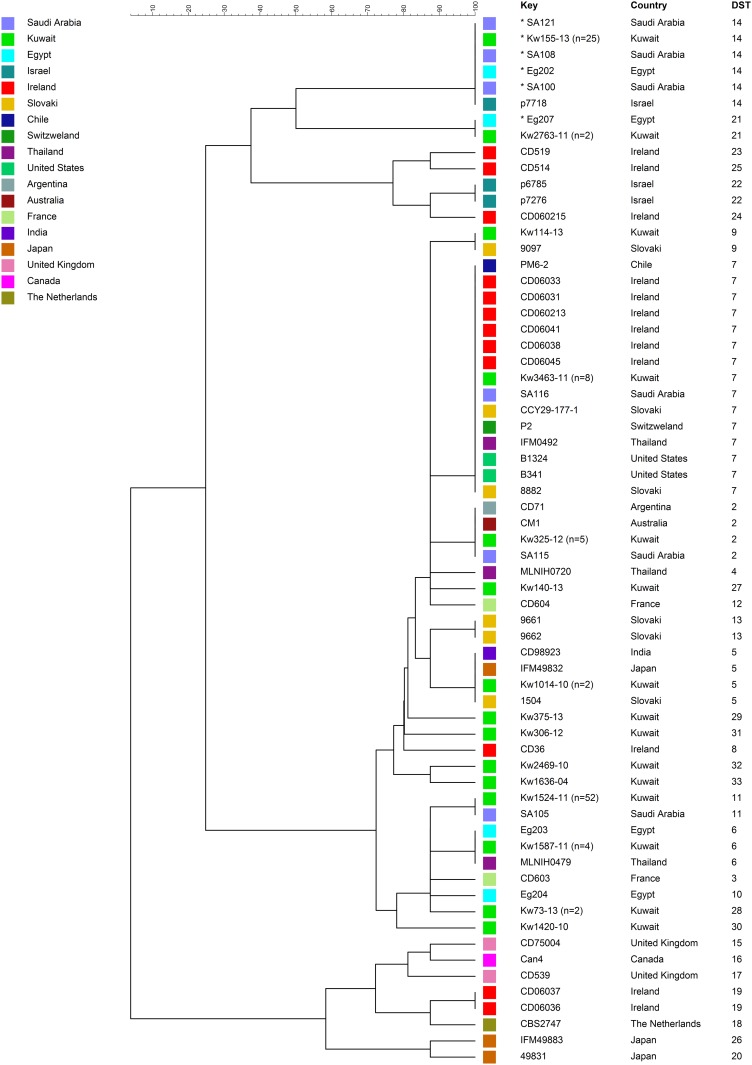
An UPGMA-derived dendrogram based on locus-specific Diploid Sequence Type (DST) of 8 housekeeping gene fragments from one representative *C*. *dubliniensis* isolate from Kuwait for each genotype together with the data from a global collection of 50 epidemiologically distinct *C*. *dubliniensis* isolates analyzed previously. Similarity is presented in percentages using the scale bar in the upper left corner and the country of isolation of the isolates are color coded. The columns from left to right include, isolate number, country of isolation and MLST-based DST. The 5-FC-resistant isolates are indicated by an asterisk (*) before the isolate number and the numbers in parenthesis indicate the number of isolates from Kuwait with the identical DST.

The genetic diversity of *C*. *dubliniensis* isolates in Kuwait was further studied by comparing the MLST-based DST data obtained in this study with the DST data generated previously [20] from a global collection of 50 epidemiologically unrelated *C*. *dubliniensis* isolates to generate a global dendrogram and the results are shown in Fig 1. For clarity of presentation, data from only one isolate were used as representative for DSTs that were shared among multiple isolates from Kuwait considering that the previous study was also based on only 50 epidemiologically unrelated *C*. *dubliniensis* isolates chosen from a global collection that included many more *C*. *dubliniensis* isolates from around the world [20]. A total of 102 of 110 *C*. *dubliniensis* isolates from Kuwait belonged to nine previously described DSTs. The two largest clusters of 52 and 25 isolates belonged to DST11 and DST14, respectively. *C*. *dubliniensis* strains belonging to these two genotypes (DST11 and DST14) were isolated only from Middle Eastern countries. The DST14 contained all (n = 25) 5-FC-resistant isolates from Kuwait but also included three 5-FC-resistant isolates from Saudi Arabia, one 5-FC-resistant isolate from Egypt and, more importantly, one 5-FC-susceptible isolate from Israel. Furthermore, two ITSH5 isolates from Kuwait that were susceptible to 5-FC belonged to MLST-based DST21. This genotype (DST21) also included a 5-FC-resistant isolate (Eg207) with similar ITS region sequence (ITSH5) from Egypt. The DST21 was related to DST14 in the phylogenetic tree (Fig 1). The *C*. *dubliniensis* isolates belonging to the remaining six DSTs (DST2, DST4, DST5, DST6, DST7 and DST9) exhibited a wider geographic distribution as other isolates with identical DSTs were obtained from one to several other distantly located countries. Of the seven newly described DSTs from Kuwait, *C*. *dubliniensis* isolates belonging to DST27 and DST28 exhibited relatively close genotypic relationship with isolates obtained from diverse geographical locations and belonging to several DSTs (Fig 1). On the contrary, five isolates belonging to the remaining five new DSTs (DST29 to DST33) from Kuwait were distinct strains suggesting that these genotypes may have a more restricted global distribution (Fig 1).

## Discussion

There were three main goals of the present study: (i) to determine whether ITS haplotypes identified in our previous study among *C*. *dubliniensis* isolates in Kuwait are also supported by MLST data, (ii) to determine whether 5-FC-resistant *C*. *dubliniensis* isolates in Kuwait are genotypically identical or heterogeneous, and (iii) to compare our MLST data with data obtained previously from a geographically diverse collection of isolates by McManus et al. [20] for determining the genetic diversity among *C*. *dubliniensis* isolates in Kuwait.

Of the 110 *C*. *dubliniensis* isolates analyzed in this study, 80 (73%) isolates belonged to ITSH1, 25 (23%) belonged to ITSH4, two isolates each belonged to ITSH5 and ITSH8 and one isolate belonged to ITSH7. The ITSH7 isolate (Kw106-08) together with two ITSH1 isolates (Kw106-08 and Kw1148-10) belonged to DST4, a genotype that was previously assigned to an isolate (MLNIH0720) from Thailand [20]. The remaining four *C*. *dubliniensis* isolates belonging to new ITS-based haplotypes (ITSH5 and ITSH8) either belonged to a distinct MLST-based DST (DST21) which included isolates from Kuwait and another Middle Eastern country (Egypt) or belonged to new DSTs (DST32 and DST33) which have not been described previously. These findings support the assignment of additional ITS-based haplotypes detected among *C*. *dubliniensis* isolates in Kuwait. Furthermore, all 25 ITSH4 isolates (including 15 isolates detected among 58 isolates analyzed in this study and 10 isolates from the previous [21] study) were resistant to 5-FC, belonged to only one MLST-based genotype (DST14) and contained S29L mutation in the Cd*FCA1*. All other (n = 85) *C*. *dubliniensis* isolates (including 43 of 58 isolates analyzed in this study) were susceptible to 5-FC and contained wild-type sequence at Cd*FCA1* codon 29. On the contrary, 5-FC-resistant clinical *C*. *dubliniensis* isolates from two other Middle Eastern countries (Saudi Arabia and Egypt) were distributed across both, ITSH3 and ITSH4 while isolates belonging to these two haplotypes from other geographical locations were susceptible to 5-FC and contained wild-type sequence at Cd*FCA1* codon 29 [18, 19, 30]. Furthermore, the two isolates (Kw256/06 and Kw2763/11) belonging to ITSH5 and DST21 were susceptible to 5-FC. An isolate from Egypt with similar ITS sequence (Eg207) and also belonging to DST21 was previously reported as resistant to 5-FC [19, 30].

Among antifungal drugs, triazoles (such as fluconazole, itraconazole, voriconazole, posaconazole and isavuconazole), echinocandins (such as caspofungin, anidulafungin and micafungin) and, to a lesser extent, polyenes (such as amphotericin B) are mostly used for invasive *Candida* infections while 5-FC is sparingly used around the world including Kuwait [31, 32]. *Candida* species exhibit varying degrees of *in vitro* susceptibility to these antifungal drugs. While a few recognized cases of intrinsic resistance of some species to specific antifungal agents exist, resistance to triazoles and echinocandins usually develops in a stepwise manner during prolonged therapy as a result of induced changes and mutations [31–33]. All 5-FC-resistant *C*. *dubliniensis* isolates described so far from various Middle Eastern countries including Kuwait contain a single point mutation (S29L) in the Cd*FCA1* gene [21, 30]. All (n = 25) 5-FC-resistant *C*. *dubliniensis* isolates from Kuwait belonged to DST14. However, DST14 isolates from other Middle Eastern countries exhibit variable susceptibility to 5-FC. Among five *C*. *dubliniensis* isolates belonging to DST14, three isolates from Saudi Arabia (SA100, SA108 and SA121) and one isolate from Egypt (Eg202) were resistant to 5-FC while one isolate from Israel (p7718) was susceptible to 5-FC [19, 20, 30]. Furthermore, two isolates (Kw256-06 and Kw2763-11) from Kuwait belonging to a genotype related to DST14 (DST21) were susceptible to 5-FC while one DST21 isolate from Egypt (Eg207) was resistant to 5-FC [19–21, 30].

The presence of a single point mutation in Cd*FCA1* conferring resistance to 5-FC in some isolates belonging to two distinct genotypes (DST14 and DST21) suggests intrinsic resistance. The resistance to 5-FC in *C*. *dubliniensis* originated in the Middle East either in the common ancestor of DST14 and DST21 lineages and was subsequently lost in some isolates or the resistance originated independently in multiple lineages of *C*. *dubliniensis* strains. Fingerprinting of these 5-FC-resistant isolates and inclusion of additional isolates from other Middle Eastern countries by more discriminatory methods (such as whole genome sequencing) will be required to confirm or exclude these possibilities. In the sister species, *C*. *albicans*, 5-FC-resistant isolates also belong to several MLST clades. Nearly 73% of 5-FC-resistant *C*. *albicans* isolates belong to Clade 1 while 27% of 5-FC-resistant isolates belong to six other clades (Clade 2, Clade 5, Clade 6, Clade 11, Clade 12 and Clade 17) [34, 35]. On the contrary, resistance to triazoles is more evenly distributed across different clades [35]. These findings and the rare use of 5-FC as an antifungal drug support the intrinsic nature of resistance of *C*. *dubliniensis* to 5-FC as the resistance does not seem to be bearing a cost to resistance. This is unlike other antifungal drugs such as triazoles, echinocandins and polyenes where resistance comes at a fitness cost to the fungus in the form of reduced replication rate, transmissibility and/or lower virulence [36]. The molecular mechanisms of resistance to fluconazole are numerous and the effect of mutations conferring fluconazole resistance on fitness are also complex with some studies reporting increased fitness while others have shown reduced fitness of *Candida* spp. under different growth conditions [37–39]. Mutations conferring resistance to echinocandins in *C*. *albicans* also appeared soon after widespread use of these antifungal drugs but these mutations are associated with fitness and virulence costs, thus limiting their epidemiological and clinical impact [40, 41]. On the other hand, emergence of resistance to amphotericin B among *Candida* spp. has been rarely reported despite >50 years of clinical use. The mutations conferring resistance to amphotericin B in rare *C*. *albicans* strains created diverse stresses in this dimorphic yeast that diminished its ability to ward off host defenses making mutant strains defective in filamentation and tissue invasion and hypersensitive to oxidative stress, febrile temperatures and neutrophil-mediated clearance of the pathogen in the host [42, 43].

The MLST scheme based on 8 recommended housekeeping genes was developed by McManus et al. in 2008 by sequence analysis of several combinations of 7–10 housekeeping gene fragments and 25 DSTs were identified among 50 epidemiologically unrelated *C*. *dubliniensis* strains [20]. However, to the best of our knowledge, no other study has performed MLST-based fingerprinting of clinical *C*. *dubliniensis* isolates after their study. Furthermore, a publicly available online curated MLST database similar to *C*. *albicans* is also not available for *C*. *dubliniensis*. For this reason, additional MLST-based DSTs of *C*. *dubliniensis* detected in this study were assigned a new and higher number considering that 26 DSTs (based on all 10 instead of 8 recommended housekeeping gene fragments) have been described previously [20]. The genetic diversity of *C*. *dubliniensis* isolates recovered from clinical specimens in Kuwait was compared to a more geographically diverse collection of 50 *C*. *dubliniensis* isolates by combining our MLST-based DST data with the DST data obtained previously by McManus et al. [20] to generate a global dendrogram. Although a vast majority (102 of 110, 93%) of *C*. *dubliniensis* isolates from Kuwait belonged to 9 previously described DSTs, most (79 of 102, 77%) isolates were restricted to only three DSTs (DST11, DST14 and DST21). Interestingly, other isolates belonging to these three genotypes were described only from three other (Egypt, Israel and Saudi Arabia) Middle Eastern countries [19, 20, 30]. Some genotypes of *C*. *albicans* have also been described with a geographical specificity for Asian countries [44, 45]. The remaining (23 of 102) *C*. *dubliniensis* isolates from Kuwait belonged to 6 other genotypes (DST2, DST4, DST5, DST6, DST7 and DST9) described previously. The dendrogram showed that these DSTs also included isolates from several other distantly located countries indicating a wider global distribution of these genotypes.

Of the seven newly described DSTs (DST27 to DST33) from Kuwait, *C*. *dubliniensis* isolates belonging to DST27 and DST28 exhibited close evolutionary relationship with isolates belonging to several DSTs and obtained from diverse geographical locations implying a wider global distribution of these genotypes. On the contrary, five isolates belonging to the remaining five new DSTs (DST29 to DST33) from Kuwait were evolutionarily distinct strains as they were distantly related with other genotypes. Our data suggest that these genotypes may have a more restricted distribution, similar to that exhibited by DST11, DST14 and DST21. Further studies from other countries including Middle Eastern countries are needed to confirm regional distribution of these new *C*. *dubliniensis* genotypes.

Although 110 *C*. *dubliniensis* isolates were recovered from both respiratory and non-respiratory clinical specimens and collected over an extended (2002 to 2013) time period in Kuwait, only 16 DSTs were detected. Our findings are thus consistent with the highly clonal nature of clinical *C*. *dubliniensis* strains as suggested by McManus et al. [20]. On the contrary, clinical isolates of the sister species (*C*. *albicans*) exhibit much greater genotypic heterogeneity as more than 3000 DSTs have been identified among >4000 *C*. *albicans* isolates analyzed from various locations around the world [1, 35, 46, http://pubmlst.org/calbicans/]. It is possible that the preferential occurrence of *C*. *dubliniensis* in specialized anatomical niches and its reduced ability to cause diverse infections in limited stress conditions in comparison to *C*. *albicans* may have had a role in conserving its clonality during the process of genetic evolution [47].

## Conclusions

The population structure of clinical *C*. *dubliniensis* isolates in Kuwait exhibited limited genotypic heterogeneity. All 5-FC-resistant *C*. *dubliniensis* isolates in Kuwait belonged to a single ITS-based haplotype (ITSH4) and a single MLST-based genotype (DST14). However, DST14 also included 5-FC-susceptible isolates from some Middle Eastern countries while one 5-FC-resistant isolate belonged to another genotype (DST21). Several other genotypes from Kuwait and other Middle Eastern countries also showed a geographical specificity.

## Supporting information

S1 Table List of 110 *C*. *dubliniensis* isolates used in this study with their ITS haplotype, Diploid Sequence Type (DST), amino acid at *CdFCA1* codon 29 and accession numbers of representative DNA sequences submitted to EMBL/GenBank databases for different ITS haplotypes/new diploid sequence types (DST27-DST33) detected in this study and for *CdFCA1* codon 29 region. (DOCX)

S2 Table Nucleotide sequences of two new locus-specific diploid sequence types determined in this study. (DOCX)
