# Supplementary material for: Population structure and molecular genetic characterization of 5-flucytosine-susceptible and -resistant clinical Candida dubliniensis isolates from Kuwait
Source: PLoS One. 2017 Apr 5;12(4):e0175269. doi: 10.1371/journal.pone.0175269 (PMC5381908; doi:10.1371/journal.pone.0175269)
Supplement: S1 Table — (DOCX) [file pone.0175269.s001.docx]

**S1 Table.** List of 110 *C. dubliniensis* isolates used in this study with their ITS haplotype, diploid sequence type (DST), amino acid at *CdFCA1* codon 29 and accession numbers of representative DNA sequences submitted to EMBL/GenBank databases for different ITS haplotypes/new diploid sequence types (DST27-DST33) detected in this study and for *CdFCA1* codon 29 region

| Serial | Isolate | Year of | Clinical | ITS | ITS sequence | MLST-based Diploid | DST sequence | *CdFCA1* | *CdFCA1* sequence | |
| --- | --- | --- | --- | --- | --- | --- | --- | --- | --- | --- |
| no. | no. | isolation | specimen^a^ | haplotype | accession no. | sequence type (DST) | accession no. | codon 29 | accession no. | |
| 1 | Kw74-02 | 2002 | Sputum | ITSH1 |  | DST11 |  | Serine | |  |
| 2 | Kw239-02 | 2002 | Vaginal swab | ITSH1 |  | DST11 |  | Serine | |  |
| 3 | Kw848-02 | 2002 | Sputum | ITSH4 |  | DST14 |  | Leucine | |  |
| 4 | Kw41-03 | 2003 | Sputum | ITSH4 | FR820636 | DST14 |  | Leucine | | FR820643 |
| 5 | Kw176-03 | 2003 | Sputum | ITSH1 |  | DST11 |  | Serine | |  |
| 6 | Kw272-03 | 2003 | Sputum | ITSH1 |  | DST6 |  | Serine | |  |
| 7 | Kw276-03 | 2003 | Vaginal swab | ITSH1 |  | DST11 |  | Serine | |  |
| 8 | Kw426-03 | 2003 | BAL | ITSH1 |  | DST11 |  | Serine | |  |
| 9 | Kw523-03 | 2003 | Tracheal aspirate | ITSH1 |  | DST7 |  | Serine | |  |
| 10 | Kw656-03 | 2003 | ETA | ITSH4 |  | DST14 |  | Leucine | |  |
| 11 | Kw672-03 | 2003 | Sputum | ITSH1 |  | DST11 |  | Serine | |  |
| 12 | Kw694-03 | 2003 | Sputum | ITSH1 |  | DST11 |  | Serine | |  |
| 13 | Kw1002-03 | 2003 | Sputum | ITSH1 |  | DST11 |  | Serine | |  |
| 14 | Kw441-04 | 2004 | Throat swab | ITSH4 |  | DST14 |  | Leucine | |  |
| 15 | Kw561-04 | 2004 | Sputum | ITSH1 |  | DST11 |  | Serine | |  |
| 16 | Kw707-04 | 2004 | ETA | ITSH1 |  | DST11 |  | Serine | |  |
| 17 | Kw759-04 | 2004 | Blood | ITSH1 |  | DST11 |  | Serine | |  |
| 18 | Kw892-04 | 2004 | ETA | ITSH1 |  | DST11 |  | Serine | |  |
| 19 | Kw994-04 | 2004 | Wound swab | ITSH1 |  | DST7 |  | Serine | |  |
| 20 | Kw1556-04 | 2004 | Sputum | ITSH1 |  | DST11 |  | Serine | |  |
| 21 | Kw1623-04 | 2004 | Vaginal swab | ITSH1 |  | DST11 |  | Serine | |  |
| 22 | Kw1632-04 | 2004 | Vaginal swab | ITSH1 |  | DST11 |  | Serine | |  |
| 23 | Kw1636-04 | 2004 | Sputum | ITSH8 |  | DST33 | LT608393 | Serine | | FR820647 |
| 24 | Kw2085-05 | 2005 | BAL | ITSH1 |  | DST5 |  | Serine | |  |
| 25 | Kw256-06 | 2006 | Sputum | ITSH5 | FR820637 | DST21 |  | Serine | | FR820644 |
| 26 | Kw1364-06 | 2006 | Sputum | ITSH4 |  | DST14 |  | Leucine | |  |
| 27 | Kw3150-07 | 2007 | Sputum | ITSH1 |  | DST6 |  | Serine | |  |
| 28 | Kw106-08 | 2008 | Sputum | ITSH7 | FR820639 | DST4 |  | Serine | | FR820646 |
| 29 | Kw3035-08 | 2008 | Sputum | ITSH1 |  | DST11 |  | Serine | |  |
| 30 | Kw3036-08 | 2008 | Sputum | ITSH1 |  | DST11 |  | Serine | |  |
| 31 | Kw3055-08 | 2008 | Sputum | ITSH1 |  | DST11 |  | Serine | |  |
| 32 | Kw3411-08 | 2008 | Sputum | ITSH1 |  | DST11 |  | Serine | |  |
| 33 | Kw275-09 | 2009 | Urine | ITSH4 |  | DST14 |  | Leucine | | KX809944 |
| 34 | Kw298-09 | 2009 | Urine | ITSH4 |  | DST14 |  | Leucine | |  |
| 35 | Kw594-09 | 2009 | Sputum | ITSH1 |  | DST2 |  | Serine | |  |
| 36 | Kw1052-09 | 2009 | Urine | ITSH1 |  | DST11 |  | Serine | |  |
| 37 | Kw1070-09 | 2009 | Sputum | ITSH1 | FN652298 | DST7 |  | Serine | |  |
| 38 | Kw1227-09 | 2009 | Sputum | ITSH4 | HE860436 | DST14 |  | Leucine | | KX809945 |
| 39 | Kw141-10 | 2010 | Sputum | ITSH1 | HE860429 | DST11 |  | Serine | |  |
| 40 | Kw355-10 | 2010 | Tracheal aspirate | ITSH1 | HE860430 | DST4 |  | Serine | |  |
| 41 | Kw654-10 | 2010 | Oral swab | ITSH1 | HE860431 | DST11 |  | Serine | |  |
| 42 | Kw782-10 | 2010 | Tracheal aspirate | ITSH1 | HE860432 | DST11 |  | Serine | |  |
| 43 | Kw792-10 | 2010 | Sputum | ITSH4 | HE860434 | DST14 |  | Leucine | |  |
| 44 | Kw816-10 | 2010 | Sputum | ITSH4 | HE860435 | DST14 |  | Leucine | | KX809946 |
| 45 | Kw843-10 | 2010 | Tracheal aspirate | ITSH1 |  | DST11 |  | Serine | |  |
| 46 | Kw1010-10 | 2010 | Sputum | ITSH1 |  | DST11 |  | Serine | |  |
| 47 | Kw1014-10 | 2010 | Tracheal aspirate | ITSH1 |  | DST5 |  | Serine | |  |
| 48 | Kw1017-10 | 2010 | Catheter tip | ITSH1 | HE860433 | DST11 |  | Serine | |  |
| 49 | Kw1144-10 | 2010 | ETA | ITSH1 |  | DST7 |  | Serine | |  |
| 50 | Kw1148-10 | 2010 | Vaginal swab | ITSH1 |  | DST4 |  | Serine | |  |
| 51 | Kw1420-10 | 2010 | Drainage fluid | ITSH1 |  | DST30 | LT608390 | Serine | |  |
| 52 | Kw1467-10 | 2010 | Tracheal aspirate | ITSH1 | HG970736 | DST11 |  | Serine | |  |
| 53 | Kw381-03 | 2003 | Vaginal swab | ITSH1 |  | DST11 |  | Serine | |  |
| 54 | Kw460-03 | 2003 | Tracheal aspirate | ITSH4 | LT608360 | DST14 |  | Leucine | | LT608386 |
| 55 | Kw647-03 | 2003 | Sputum | ITSH4 | LT608357 | DST14 |  | Leucine | | LT608378 |
| 56 | Kw1078-03 | 2003 | Urine | ITSH1 |  | DST11 |  | Serine | |  |
| 57 | Kw349-04 | 2004 | ETA | ITSH1 |  | DST11 |  | Serine | |  |
| 58 | Kw598-04 | 2004 | Sputum | ITSH1 |  | DST11 |  | Serine | |  |
| 59 | Kw676-04 | 2004 | ETA | ITSH4 | LT608361 | DST14 |  | Leucine | | LT608379 |
| 60 | Kw1630-04 | 2004 | Sputum | ITSH1 |  | DST11 |  | Serine | |  |
| 61 | Kw1820-04 | 2004 | Sputum | ITSH4 | LT608362 | DST14 |  | Leucine | | LT608380 |
| 62 | Kw323-05 | 2005 | Sputum | ITSH4 | LT608363 | DST14 |  | Leucine | | LT608381 |
| 63 | Kw331-05 | 2005 | Sputum | ITSH4 | LT608365 | DST14 |  | Leucine | | LT608383 |
| 64 | Kw350-05 | 2005 | Urine | ITSH4 | LT608366 | DST14 |  | Leucine | | LT608384 |
| 65 | Kw1282-05 | 2005 | Sputum | ITSH4 | LT608364 | DST14 |  | Leucine | | LT608382 |
| 66 | Kw1668-10 | 2010 | Catheter tip | ITSH1 |  | DST11 |  | Serine | | KX809941 |
| 67 | Kw2033-11 | 2011 | Throat swab | ITSH4 | LT608359 | DST14 |  | Leucine | | LT608385 |
| 68 | Kw2469-10 | 2010 | Drainage fluid | ITSH8 | LT608369 | DST32 | LT608392 | Serine | |  |
| 69 | Kw1376-11 | 2011 | Sputum | ITSH1 |  | DST11 |  | Serine | |  |
| 70 | Kw1387-11 | 2011 | Sputum | ITSH1 | LT608351 | DST11 |  | Serine | | KX809942 |
| 71 | Kw1433-11 | 2011 | Sputum | ITSH4 | LT608358 | DST14 |  | Leucine | | LT608377 |
| 72 | Kw1504-11 | 2011 | Sputum | ITSH1 | LT608352 | DST7 |  | Serine | |  |
| 73 | Kw1524-11 | 2011 | Tracheal aspirate | ITSH1 |  | DST11 |  | Serine | |  |
| 74 | Kw1587-11 | 2011 | Throat swab | ITSH1 |  | DST6 |  | Serine | |  |
| 75 | Kw1823-11 | 2011 | Oral swab | ITSH1 |  | DST7 |  | Serine | |  |
| 76 | Kw2613-11 | 2011 | Tracheal aspirate | ITSH1 |  | DST11 |  | Serine | |  |
| 77 | Kw2730-11 | 2011 | Urine | ITSH1 |  | DST11 |  | Serine | |  |
| 78 | Kw2763-11 | 2011 | Sputum | ITSH5 | LT608368 | DST21 |  | Serine | |  |
| 79 | Kw2942-11 | 2011 | Oral swab | ITSH1 |  | DST11 |  | Serine | |  |
| 80 | Kw2979-11 | 2011 | ETA | ITSH1 |  | DST2 |  | Serine | |  |
| 81 | Kw3326-11 | 2011 | Sputum | ITSH1 |  | DST11 |  | Serine | |  |
| 82 | Kw3329-11 | 2011 | Sputum | ITSH1 |  | DST11 |  | Serine | |  |
| 83 | Kw3463-11 | 2011 | Sputum | ITSH1 |  | DST7 |  | Serine | |  |
| 84 | Kw3471-11 | 2011 | Oral swab | ITSH1 |  | DST6 |  | Serine | |  |
| 85 | Kw3493-11 | 2011 | Sputum | ITSH1 |  | DST11 |  | Serine | |  |
| 86 | Kw3514-11 | 2011 | Tracheal aspirate | ITSH1 |  | DST11 |  | Serine | |  |
| 87 | Kw3614-11 | 2011 | Sputum | ITSH1 |  | DST11 |  | Serine | |  |
| 88 | Kw3648-11 | 2011 | Sputum | ITSH1 |  | DST28 | LT608388 | Serine | | KX809943 |
| 89 | Kw71-12 | 2012 | Sputum | ITSH1 |  | DST11 |  | Serine | |  |
| 90 | Kw72-12 | 2012 | Sputum | ITSH1 | LT608348 | DST11 |  | Serine | | LT608370 |
| 91 | Kw94-12 | 2012 | Throat swab | ITSH1 |  | DST11 |  | Serine | |  |
| 92 | Kw180-12 | 2012 | Sputum | ITSH4 | LT608354 | DST14 |  | Leucine | | LT608374 |
| 93 | Kw233-12 | 2012 | Sputum | ITSH4 | LT608355 | DST14 |  | Leucine | | LT608375 |
| 94 | Kw261-12 | 2012 | Sputum | ITSH4 | LT608356 | DST14 |  | Leucine | | LT608372 |
| 95 | Kw272-12 | 2012 | Sputum | ITSH4 | LT608367 | DST14 |  | Leucine | | LT608376 |
| 96 | Kw284-12 | 2012 | Sputum | ITSH1 | LT608349 | DST2 |  | Serine | | LT608371 |
| 97 | Kw296-12 | 2012 | Sputum | ITSH1 |  | DST11 |  | Serine | |  |
| 98 | Kw306-12 | 2012 | Drainage fluid | ITSH1 | LT608350 | DST31 | LT608391 | Serine | |  |
| 99 | Kw325-12 | 2012 | Sputum | ITSH1 |  | DST2 |  | Serine | |  |
| 100 | Kw343-12 | 2012 | Tracheal aspirate | ITSH1 |  | DST11 |  | Serine | |  |
| 101 | Kw972-12 | 2012 | Sputum | ITSH1 |  | DST11 |  | Serine | |  |
| 102 | Kw1148-12 | 2012 | Sputum | ITSH1 |  | DST11 |  | Serine | |  |
| 103 | Kw73-13 | 2013 | ETA | ITSH1 | LT716018 | DST28 |  | Serine | |  |
| 104 | Kw114-13 | 2013 | Sputum | ITSH1 |  | DST9 |  | Serine | |  |
| 105 | Kw138-13 | 2013 | Sputum | ITSH1 |  | DST7 |  | Serine | |  |
| 106 | Kw140-13 | 2013 | BAL | ITSH1 | LT716019 | DST27 | LT608387 | Serine | |  |
| 107 | Kw155-13 | 2013 | Drainage fluid | ITSH4 | LT608353 | DST14 |  | Leucine | | LT608373 |
| 108 | Kw169-13 | 2013 | BAL | ITSH1 |  | DST11 |  | Serine | |  |
| 109 | Kw325-13 | 2013 | Sputum | ITSH1 |  | DST2 |  | Serine | |  |
| 110 | Kw375-13 | 2013 | Sputum | ITSH1 | LT716020 | DST29 | LT608389 | Serine | |  |

^a^BAL, bronchoalveolar lavage; ETA, endotracheal aspirate.
