# Supplementary material for: Population structure and molecular genetic characterization of 5-flucytosine-susceptible and -resistant clinical Candida dubliniensis isolates from Kuwait
Source: PLoS One. 2017 Apr 5;12(4):e0175269. doi: 10.1371/journal.pone.0175269 (PMC5381908; doi:10.1371/journal.pone.0175269)
Supplement: S2 Table — (DOCX) [file pone.0175269.s002.docx]

**S2 Table.** Nucleotide sequences of two new locus-specific diploid sequence types determined in this study

**>*CdAAT1b*-6**

TTAACTAAATTGGCTAATGAAAATAAAATCCCTTCATTTGCTTTGTGTCAATCATTTGCTAAAAATATGGGACTTTATGGAGAAAGAACTGGATCAATTTCTATTATTAATTCATCGAGTGAAGATTCTAAAGCAGTTGAATCTCAATTGAAAAAATTAATTAGACCAATTTATTCTTCTCCACCAATTCATGGATCKAAAATTGTTGAAATTATTTTTGATGAAAATTCTGGTTTATTACCTCAATGGTTAGATGAATTAGATAAAGTTGTTGGAAGATTAAATACTGTTCGTTCTAAATTATATGAAAAATTAGATAAATCTAATTATAATTGGGATCA

**>*CdMPIb*-8**

TTTAAGCCTTTGGATCAATTGGCTAAAACTTTGACTACAGTTCCTGAATTAAACGAAATTATTGGTCAAGAATTGGTTGATGAATTTGTTAGTGGTATTARACTACCAGCAGAAATTGGAAGTCAAGATGATGTTAACAATAGAAAATTGTTACAAAAAGTGTTTGGTAAATTAATGAACACCAATGAAGATATTATAAAGCAACAAACTACTAAATTACTTGAAAGAACAGAAAGGGAACCTCAAGTGTTTAAAAACATTGATTCTAGATTACCAGAGTTAATACAAAGATTGAACAAGCAATTTCCTAATGATATTGGATTATTTTGTGGATGTCTCTTGTTGAACCACGTTGGCTTGAACAAAGGGGAAGCG
